# A Set of Intein‐Split Selectable Marker Genes for Efficient Co‐Transformation

**DOI:** 10.1111/pbi.70502

**Published:** 2025-12-16

**Authors:** Fabio G. Moratti, Chiara Lonoce, Stephan Obst, Xenia Kroop, Daniel Karcher, Stephanie Ruf, Ralph Bock

**Affiliations:** ^1^ Max‐Planck‐Institut für Molekulare Pflanzenphysiologie (MPI‐MP) Potsdam‐Golm Germany

**Keywords:** co‐transformation, hygromycin resistance, intein, kanamycin resistance, protein splicing, selectable marker gene, split marker gene, sulfadiazine resistance

Technologies for plant genetic engineering are critically dependent on marker genes that allow the selection of transgenic cells. Multigene engineering and transgene stacking by co‐transformation or supertransformation often require the use of multiple selectable marker genes. However, only a limited number of efficient marker genes are available (Liu et al. 2013; Tabatabaei et al. 2019), and for the transformation of many plant species, a single marker gene is strongly preferred because of its superior efficiency. This limitation in multigene engineering can be alleviated by employing split selectable markers that enable the simultaneous introduction of two transformation vectors using only a single selection agent (Palanisamy et al. 2019). In addition, split markers are useful tools to overcome vector size limits, enable modular combinatorial testing of constructs, and facilitate trait stacking via sexual crosses. A useful strategy for marker splitting relies on protein *trans*‐splicing, in which the marker gene fragments (placed on two separate vectors) are fused to fragments encoding the N‐terminal or C‐terminal half of an intein. When both vectors are introduced into a target cell, expression of the two marker protein‐intein fragments leads to the removal of the intein by *trans*‐splicing and reconstitution of the full‐length marker protein, thus conferring the desired resistance. This approach has been successfully used in bacteria and mammalian cell cultures (Palanisamy et al. 2019; Jillette et al. 2019), but has not been widely employed in plant transformation. A recent report described a split kanamycin resistance gene and a split hygromycin resistance gene, but the efficiency of the split genes compared to the unsplit marker has not been assessed (Yuan et al. 2023).

Here, we have taken a systematic approach and constructed several intein‐split selectable markers and evaluated their efficiencies in plant transformation. We split three commonly used markers: the antibiotic resistance genes *hpt* (conferring hygromycin resistance) and *nptII* (conferring kanamycin resistance), and the herbicide resistance gene *sul* (conferring sulfadiazine resistance; Figure 1a; Figure S1a). While the split sites for *hpt* and *nptII* were based on previous studies (Jillette et al. 2019; Yuan et al. 2023), a suitable split site for *sul* was identified based on the analysis of the three‐dimensional protein structure (Figure 1a). The N‐terminal fragment of each marker gene was translationally fused to the N‐terminal fragment of the *Npu*DnaE intein. In a separate plasmid vector, the C‐terminal fragment of the intein was fused to the C‐terminal fragment of the marker (Figure 1a; Jillette et al. 2019). As controls, plasmid vectors containing the full‐length marker genes under identical expression elements were constructed.

**FIGURE 1:**
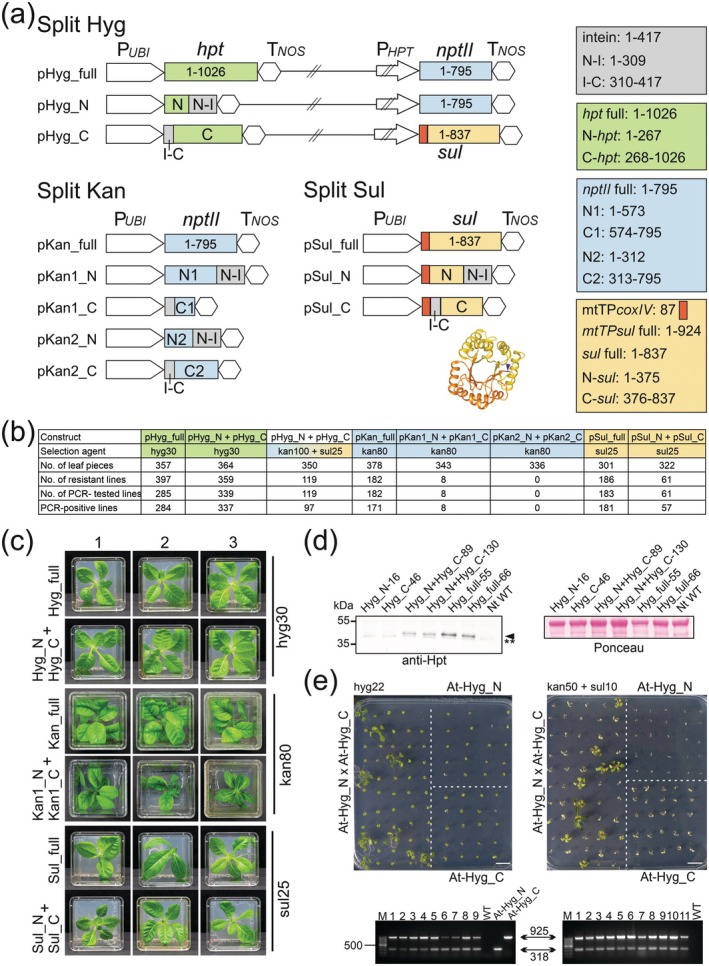
Evaluation of intein‐split marker genes for plant transformation. (a) Design of transformation vectors for the analysis of split hygromycin (Hyg), kanamycin (Kan) and sulfadiazine (Sul) genes. Vector elements not drawn to scale are indicated by diagonal double strikethrough. The newly designed split site in the Sul protein is indicated in the three‐dimensional structure (blue arrowhead). (b) Transformation efficiencies obtained with the various constructs in tobacco. (c) Antibiotic resistance of transgenic tobacco plants expressing co‐transformed intein‐split marker gene constructs in comparison to plants expressing the full‐length marker genes. (d) Immunoblot analysis of Hpt protein accumulation in transgenic tobacco plants. Two lines obtained by co‐transformation of the intein‐split marker constructs and two lines generated by transformation with the full‐length *hpt* are shown. As controls, the wild type and transgenic lines expressing only the N‐terminal or the C‐terminal Hpt fragment are included. The arrowhead marks the Hpt signal, the double asterisks a cross‐reaction (serving as loading control, in addition to the Ponceau staining). (e) Combination of intein‐split *hpt* gene fragments by genetic crosses in *Arabidopsis*. The photographed plates show the Mendelian segregation of the progeny from the cross of a hemizygous At‐Hyg_N plant with a hemizygous At‐Hyg_C plant. Seeds were sown on either hygromycin‐containing medium (to select for reconstitution of the split Hpt marker; left) or medium containing both kanamycin and sulfadiazine (the markers used to generate the transgenic lines expressing the split fragments; right). The ethidium‐bromide‐stained agarose gels show PCR assays (with three primers each; see Methods) confirming the presence of the marker gene fragments in the transgenic lines obtained from the selection plates shown above. Amplicon sizes and the 500 bp marker fragment are indicated.

First, the two vectors containing the split hygromycin marker (pHyg_N and pHyg_C; Figure 1a) were introduced into tobacco ( *Nicotiana tabacum* ) leaf cells by biolistic co‐transformation, and compared to transformation with the vector containing the full‐length *hpt* (pHyg_full). Selection on hygromycin‐supplemented medium and genotyping of resistant regenerants by PCR (Supporting Information Methods; Tables S1–S3) resulted in 284 transgenic lines obtained with the full‐length marker and 337 transgenic lines obtained with the split marker (Figure 1b,c). These numbers indicate that the split *hpt* marker is as efficient as the full‐length marker. Immunoblot analysis with anti‐Hpt antibodies confirmed efficient protein *trans*‐splicing and accumulation of the ligation product in hygromycin‐resistant plants (Figure 1d).

Next, we used the same approach to evaluate the split kanamycin resistance gene *nptII*. Surprisingly, co‐transformation of the intein‐split constructs (pKan1_N and pKan1_C) produced only 8 kanamycin‐resistant lines compared to 171 resistant lines obtained with the unsplit construct pKan_full (Figure 1b,c). This very low efficiency of the split *nptII* was surprising, given that the same split site was used in the only previous report on intein‐split markers in plants (Yuan et al. 2023). When we tested a different split site (pKan2_N and pKan2_C; Figure 1a), no transgenic lines were obtained (Figure 1b), suggesting that the *nptII* gene is less suitable for marker splitting than *hpt*.

To provide an alternative to *hpt*, we engineered an intein‐split *sul* (Figure 1a), encoding a mutated (sulfadiazine‐resistant) dihydropteroate synthase. Since Sul function requires mitochondrial localisation (Tabatabaei et al. 2019), both intein‐split *sul* fragments were tethered to a mitochondrial targeting sequence (pSul_N and pSul_C; Figure 1a). Co‐transformation experiments and control transformations with the full‐length marker (pSul_full) followed by selection on sulfadiazine‐supplemented medium and PCR genotyping yielded 57 transgenic lines with the split marker and 181 with the full‐length marker (Figure 1b,c). Taken together, these data indicate that the intein‐split *hpt* provides the most efficient system, in that it provides high transformation efficiencies that rival those of the full‐length control. Our newly designed intein‐split *sul* may be slightly less efficient, while the split *nptII* currently cannot be recommended for plant transformation.

To assess whether the efficiency of the three tested split marker systems was related to the efficiency of protein *trans*‐splicing, we examined the splicing efficiency of the intein‐split fragments by recombinant expression in *E. coli* (Figure S1a). *E. coli* cells transformed with vectors co‐expressing the two intein‐split marker gene fragments grew on media supplemented with the corresponding selection agent in all three cases (Figure S1b). However, colonies expressing the intein‐split *nptII* were substantially smaller in size, suggesting low‐level resistance to kanamycin. Poor performance of the split *nptII* and high efficiency of the split *hpt* were further confirmed by comparative growth assays in liquid medium under antibiotic selection (Figure S2). When the accumulation of the mature *trans*‐spliced protein forms was assessed by Coomassie staining, high levels of mature Hpt were detected, but very low levels of mature NptII protein (Figure S1c), suggesting poor splicing as the reason for the very low efficiency of the split *nptII*. Accumulation of spliced Sul was also low, but apparently is sufficient to confer full resistance in bacteria and provide good performance in plants.

In addition to the intein‐split *hpt* fragments, the pHyg_N and pHyg_C vectors harboured a full‐length marker gene: *nptII* in pHyg_N and *sul* in pHyg_C, to also facilitate selection independent of Hpt splicing (Figure 1a). This set‐up allowed us to compare the selection efficiency on hygromycin with that on double selection for kanamycin and sulfadiazine. The double selection turned out to be significantly less efficient than the hygromycin selection based on the split *hpt*, both with respect to the total number of transgenic lines obtained and the background of escapees that did not harbour the transgenes (Figure 1b). These data suggest that the split marker approach is superior to co‐transformation with two selectable marker genes.

Finally, we evaluated the possibility of combining the two marker gene fragments by sexual crosses in the model plant *Arabidopsis thaliana* . To this end, we generated stably transformed *Arabidopsis* plants with vectors pHyg_N (At‐Hyg_N lines) and pHyg_C (At‐Hyg_C lines). The At‐Hyg_N lines were then crossed to the At‐Hyg_C lines, and the resulting F1 seeds were sown on hygromycin‐containing medium. This resulted in the selection of hygromycin‐resistant progeny at the expected Mendelian segregation ratio (Figure 1e). All resistant seedlings contained both the pHyg_N and the pHyg_C transgenes, as assayed by PCR (Figure 1e). This experiment indicates that intein‐split markers can be reconstituted also by genetic crosses, and plants containing two transformation constructs can be efficiently identified by using only a single selection agent.

In summary, our work reported here provides two efficient intein‐split markers for plant transformation. The intein‐split *hpt* appears to be the most efficient split marker, both in terms of transformation efficiency and protein *trans*‐splicing efficiency. Our newly engineered intein‐split *sul* provides a viable alternative, while the split *nptII* cannot be recommended.

## Author Contributions

F.G.M., C.L., D.K., S.R. and R.B. designed the research. F.G.M., C.L., S.O., X.K. and D.K. performed the experiments. All authors participated in data evaluation. F.G.M., C.L., S.R. and R.B. wrote the manuscript, with input from all co‐authors.

## Funding

This work was supported by H2020 European Research Council, ERC‐ADG‐2014 (Grant 669982), Max‐Planck‐Gesellschaft.

## Supporting information


**Figure S1–S2**.
**Table S1–S3**.

## Data Availability

The data that support the findings of this study are available in the manuscript and its associated Supporting Information.

## References

[pbi70502-bib-0001] Jillette, N. , M. Du , J. J. Zhu , P. Cardoz , and A. W. Cheng . 2019. “Split Selectable Markers.” Nature Communications 10: 4968. 10.1038/s41467-019-12891-2 PMC6823381 31672965

[pbi70502-bib-0002] Liu, W. , J. S. Yuan , and C. N. Stewart Jr. 2013. “Advanced Genetic Tools for Plant Biotechnology.” Nature Reviews Genetics 14: 781–793. 10.1038/nrg3583 24105275

[pbi70502-bib-0003] Palanisamy, N. , A. Degen , A. Morath , et al. 2019. “Split Intein‐Mediated Selection of Cells Containing Two Plasmids Using a Single Antibiotic.” Nature Communications 10: 4967. 10.1038/s41467-019-12911-1 PMC6823396 31672972

[pbi70502-bib-0004] Tabatabaei, I. , C. Dal Bosco , M. Bednarska , S. Ruf , J. Meurer , and R. Bock . 2019. “A Highly Efficient Sulfadiazine Selection System for the Generation of Transgenic Plants and Algae.” Plant Biotechnology Journal 17: 638–649. 30144344 10.1111/pbi.13004 PMC6381783

[pbi70502-bib-0005] Yuan, G. , H. Lu , K. De , et al. 2023. “Split Selectable Marker Systems Utilizing Inteins Facilitate Gene Stacking in Plants.” Communications Biology 6: 567. 37237044 10.1038/s42003-023-04950-8 PMC10219933

