# Supplementary material for: A Set of Intein‐Split Selectable Marker Genes for Efficient Co‐Transformation
Source: Plant Biotechnol J. 2025 Dec 16;24(4):2439–41. doi: 10.1111/pbi.70502 (PMC13140669; doi:10.1111/pbi.70502)
Supplement: Supplementary file 1 — Figure S1–S2. Table S1–S3. [file PBI-24-2439-s001.pdf]

SUPPLEMENTARY INFORMATION

Experimental procedures

Plant material and growth conditions. For transformation experiments in tobacco (*Nicotiana tabacum*), the cultivar Petit Havana was used. For cultivation under aseptic conditions, surface-sterilized seeds were germinated on synthetic medium (MS medium; Murashige and Skoog, 1962) supplemented with 3% sucrose. Plants were grown in a 16-hour photoperiod at a light intensity of 55 µmol photons m^{-2} s^{-1} and a temperature of 25°C, followed by an 8-hour dark period at 20°C. Selection and regeneration experiments were performed under similar conditions, except that the light intensity was reduced to 25 µmol photons m^{-2} s^{-1}. Plant growth in soil was conducted under standard greenhouse conditions.

For transformation of *Arabidopsis thaliana*, ecotype Col-0 was used. Plants were raised from seeds, and after germination, grown for one week in long-day conditions (in a 16 h light/8 h dark cycle), and then continued to grow under short-day conditions (8 h light/16 h dark cycle). The growth light intensity was 120 µmol photons m^{-2} s^{-1}, and the temperature was 21°C during the day and 19°C at night.

Construction of transformation vectors. The split point for the *hpt* gene (Y89:C90) and the initial split point for the *nptII* marker (A191:C192) were chosen based on a previous study (Jillette et al., 2019). To identify a suitable split site in the Sul protein, we evaluated all cysteine, serine and threonine residues for their suitability to serve as catalytic residues for intein splicing, and discarded those that are upstream of residues considered to be incompatible with efficient intein splicing (Cheriyan et al., 2013). Next, we discarded those putative split points that are too close to the N-terminus or the C-terminus of the protein and may not divide the protein into two catalytically inactive fragments (based on the active site of the Sul enzyme; Baca et al., 2000). From the remaining candidate sites, we chose the split point H125:S126, because it resides in a surface-exposed, low-complexity loop.

The intein-split marker fragments were translationally fused to the corresponding N-terminal or C-terminal fragments of the well characterized *Npu*DnaE

intein from *Nostoc punctiforme* (Cheriyan et al., 2013; Figure 1). The intein fragments are referred to as N-I and I-C. All vectors contain a full-length selectable marker gene and either the N-terminal or the C- terminal fragment of the split marker (Figure 1a). All intein-split marker fragment fusions were codon optimized for the preferred codon usage in the tobacco nuclear genome, placed between the promoter of the ubiquitin 10 gene (P_{UBI}) and the *nos* terminator (T_{NOS}), and the resulting sequences were chemically synthesized (ThermoFisher). The synthesized fragments were introduced in the intended recipient vector by InFusion cloning (Clontech Laboratories, Mountain View, CA) as follows. Vectors pHyg_N, pSul_N, pHyg_full, and pSul_full were obtained by introducing the synthetic fragments P_{UBI}::*hpt*(1-89)::N-I::T_{NOS}, P_{UBI}::TP_{coxIV}::*sul*(1-125)::N-I::T_{NOS}, P_{UBI}::*hpt*::T_{NOS} and P_{UBI}::TP_{coxIV}::*sul*::T_{NOS}, respectively, into vector pDK318 digested with the restriction enzymes NheI and ApaI. pDK318 is a binary vector derived from pORE_R3 (Coutu et al., 2007), and modified by insertion of a GFP expression cassette into the SmaI/EcoRV-digested pORE_R3. The *GFP* is driven by the CaMV 35S promoter and fused to the *RBCS* transit peptide sequence from *Arabidopsis*. To generate pKan2_N and pSul_C, the synthetic sequences P_{UBI}::*nptII*(1-263)::N-I::T_{NOS} and P_{UBI}::I-C::*sul*(126-278)::T_{NOS} were introduced into pCL20 digested with NheI and ApaI. pCL20 is a derivative of pIT35 (Tabatabaei et al., 2019) that carries the *hpt* selectable marker gene instead of *sul.* Vectors pHyg_C, pKan2_C and pKan_full were obtained by introducing the synthetic fragments P_{UBI}::I-C::*hpt*(90-341)::T_{NOS}, P_{UBI}::I-C::*nptII*(105-264)::T_{NOS} and P_{UBI}::*nptII*::T_{NOS}, respectively, into pIT35 digested with NheI and ApaI. To create pKan1_N, the synthesized fragment *nptII*(1-191)::N-I was introduced into pKan_full digested with NheI and SpeI. pKan1_C was obtained by introducing the synthetic fragment P_{UBI}::I-C::*nptII*(192-265)::T_{NOS} into vector pLD10 linearized with SfoI. pLD10 is a binary vector similar to pDK318, but derived from pORE_E4 (Coutu et al., 2007) and carrying the *hpt* selectable marker gene (driven by the CaMV 35S promoter and inserted into the AscI/FseI-digested vector).

All plasmids for recombinant protein expression in the Rosetta strain of *Escherichia coli* were based on vector pETDuet-1 (Novagen). To produce pET_Hyg_N, a PCR product generated by amplification of the *hpt*(1-89)::N-I sequence from vector pHyg_N with primer pair oFGM439/oFGM440 (Supplementary Table 1) was introduced in pETDuet-1 digested with NcoI and HindIII by InFusion cloning.

Similarly, pET_Hyg_C resulted from cloning the PCR product obtained by amplification of I-C::*hpt*(90-341) from pHyg_C using primers oFGM441 and oFGM442 into pETDuet-1 digested with NdeI and PacI. The same PCR product was introduced into pET_Hyg_N digested with NdeI and PacI to generate pET_Hyg_NC, harboring both intein-split *hpt* fragments (Supplementary Figure 1). pET_Sul_N was generated by introducing the PCR fragment obtained by amplification of pSul_N with primer pair oFGM574/oFGM575 into pETDuet-1 digested with NcoI/HindIII, and pET_Kan_N was produced by introducing the PCR product obtained with primers oFGM585 and oFGM440 into the same vector. pET_Sul_C was generated by cloning the PCR fragment obtained from amplification of pSul_C with primer pair oFGM576/oFGM577 into pETDuet-1 digested with NdeI and PacI, and pET_Kan_C was produced by introducing the PCR product obtained by amplification of pKan_C with primer pair oFGM441/oFGM587 into the same vector. pET_Kan_NC was generated by introducing the PCR product obtained by using pKan_C as template and primer pair oFGM441/oFGM587 into pET_Kan_N digested with NdeI and PacI. pET_Sul_NC was produced by introducing the PCR product obtained by using pSul_C as template and primer pair oFGM576/oFGM577 into pET_Sul_N digested with NdeI and PacI. To create pET_Kan_full, the full-length *nptII* gene was amplified from pKan_full with primer pair oFGM585/oFGM586 and introduced in pETDuet-1 digested with NcoI and HindIII. Finally, to generate pET_Sul_full, the full-length *sul* coding region was amplified from pSul_full with primer pair oFGM583/oFGM584 and introduced in pETDuet-1 digested with NcoI and HindIII (Supplementary Table1; Supplementary Figure 1).

Plant transformation. Biolistic transformation experiments in tobacco were carried out using a BioRad PDS-1000/He biolistic gun equipped with a hepta adapter. Young leaves of aseptically grown plants raised on agar-solidified MS medium were bombarded with vector DNA-coated 0.6 µm gold particles (Bio-Rad) following published protocols (Ruf and Bock, 2021). The bombarded leaves were then cut into 5 × 5 mm pieces, and cultured on plant regeneration medium (Ruf and Bock, 2011) supplemented with the appropriate selection agent. Resistant shoots were transferred to

phytohormone-free medium and, after rooting, transferred to soil and grown under standard greenhouse conditions.

Agrobacterium-mediated transformation of *Arabidopsis* plants was performed by the floral dip method (Clough and Bent, 1998) and the resulting seeds were sown on half-strength MS medium containing 1% sucrose and the appropriate selection agent. Plants transformed with pHyg_N were selected on 50 mg/L kanamycin, and plants transformed with pHyg_C were selected on 10 mg/L sulfadiazine. Resistant plants containing the Hyg_N-encoding cassette were crossed to plants containing the pHyg_C-encoding cassette, and the resulting F1 seeds were subjected to assays for intein-split protein reconstitution by plating on MS medium supplemented with 22 mg/L hygromycin. For comparison, seeds obtained from the same cross were also sown on MS medium supplemented with 10 mg/L sulfadiazine plus 50 mg/L kanamycin.

Plant genotyping. For genotyping, total genomic DNA was extracted from fresh leaf tissue using a CTAB-based protocol (Doyle and Doyle, 1990). Plants co-transformed with pHyg_C and pHyg_N were genotyped using a PCR mix containing the three primers oFGM523, oFGM442 and oFGM444 (Supplementary Tables 2 and 3). Presence of pHyg_full was assayed with primer pair oFGM523/oFGM444. Plants co-transformed with pKan_C and pKan_N were genotyped using a PCR mix containing the three primers oFGM523, oFGM533 and oFGM578. Presence of pKan_full was tested with primer pair FGM523/oFGM533. Plants co-transformed with pSul_C and pSul_N were genotyped using a PCR mix containing the three primers oFGM523, oFGM520 and oFGM521. Presence of pSul_full was tested with primers oFGM523 and oFGM552 (Supplementary Tables 2 and 3).

Analysis of protein splicing. Competent cells of *Escherichia coli* Rosetta BL21(DE3) pLysS were transformed by the standard heat-shock method with pETDuet-1-based expression constructs (see above; Supplementary Figure 1), and transgenic colonies were selected on agar-solidified LB medium supplemented with ampicillin (50 µg/mL). Single colonies were inoculated in 25 mL of LB medium supplemented with ampicillin, grown at 37°C at 180 rpm to an OD_{600} of 0.25, and then transferred to 28°C. At an OD_{600} of 0.5, cells were induced with 0.5 mM IPTG. Cell pellets were harvested 5 h

after induction by centrifugation, resuspended in 1% SDS and centrifuged for 15 min at 14,000 *g*. The supernatants were mixed with loading buffer for SDS-PAGE, and electrophoretically separated at 100 V.

Antibiotic sensitivity assays in bacteria. To test *E. coli* Rosetta strains expressing the various marker gene constructs for their resistance to kanamycin or hygromycin, single colonies were inoculated and grown in 25 mL LB medium with ampicillin (50 µg/mL). At OD_{600} of 0.5, cells were induced with 0.5 mM IPTG for 1 h, and samples of 180 µL were then plated on solid LB medium supplemented with 0.5 mM IPTG, 50 µg/mL ampicillin and either kanamycin (50 µg/mL) or hygromycin (200 µg/mL). The plates were incubated overnight at 37°C and then photographed. Resistance to sulfadiazine was tested similarly, with two differences: (i) M9 minimal medium was used instead of LB medium, and (ii) the induced cells were plated on M9 minimal medium supplemented with 0.5 mM IPTG, 50 µg/mL ampicillin and 20 µg/mL sulfadiazine.

For growth assays in liquid culture, bacterial strains were grown overnight in LB medium with ampicillin (50 µg/mL). A 1:1000 dilution was then inoculated in fresh medium containing ampicillin (50 µg/mL) and IPTG (0.5 mM), and cells were grown to an OD_{600} of about 0.05 at 28°C. Afterwards, the appropriate antibiotic was added, and bacterial growth was monitored for 6 hours by measuring the OD_{600} every 40 minutes.

Immunodetection of the mature Hpt protein. To confirm the accumulation of mature, spliced Hpt protein in tobacco plants, samples of 20 µg total protein extracted from leaves were resolved by electrophoresis in 12% SDS-PAA gels, and blotted onto nitrocellulose membranes with a pore size of 0.2 µm (Cytiva Amersham™ Protran™ NC). After Ponceau-S staining and blocking with 3% non-fat milk and 0.5% BSA, the membranes were analyzed by immunodecoration with anti-HPT antibodies (PHY0625, PhytoAB, 1:2000) and HRP-conjugated goat anti-mouse secondary antibodies (Agrisera, AS11 1772, 1:25000).

Supplementary References

Baca, A.M., Sirawaraporn, R., Turley, S., Sirawaraporn, W. and Hol, W.G. (2000) Crystal structure of Mycobacterium tuberculosis 7,8-dihydropteroate synthase in complex with pterin monophosphate: new insight into the enzymatic mechanism and sulfa-drug action. *J. Mol. Biol.,* **302**, 1193-1212.

Cheriyan, M., Pedamallu, C.S., Tori, K. and Perler F. (2013) Faster protein splicing with the Nostoc punctiforme DnaE intein using non-native extein residues. *J. Biol. Chem.,* **288**, 6202-6211.

Clough, S.J. and Bent, A.F. (1998) Floral dip: a simplified method for Agrobacterium-mediated transformation of Arabidopsis thaliana. *Plant J.* **16**, 735-743.

Coutu, C., Brandle, J., Brown, D., Brown, K., Miki, B., Simmonds, J. and Hegedus, D.D. (2007) pORE: a modular binary vector series suited for both monocot and dicot plant transformation. *Transgenic Res.* **16**, 771-781.

Doyle, J.J. and Doyle, J.L. (1990) Isolation of plant DNA from fresh tissue. *Focus* **12**, 13-15.

Murashige, T. and Skoog, F. (1962) A revised medium for rapid growth and bio assays with tobacco tissue culture. *Physiol. Plant.,* **15**, 473-497.

Ruf, S. and Bock, R. (2011) In vivo analysis of RNA editing in plastids. *Meth. Mol. Biol.,* **718**, 137-150.

Ruf, S. and Bock, R. (2021) Plastid transformation in tomato: a vegetable crop and model species. *Meth. Mol. Biol.,* **2317**, 217-228.

Supplementary Tables and Figures

Supplementary Table 1. List of oligonucleotides used for cloning. Stretches of sequence homology to the target gene, which were used for InFusion seamless cloning, are underlined. N-I, *Npu*DnaE intein N-terminus, I-C, *Npu*DnaE intein C-terminus.

**Name**	**Sequence (5´ → 3´)**	**Use**
oFGM439	AGGAGATATACCATGAAAAAGCCTGAACTTACTGC	*hpt*(1-89)::N-I insert, FW (InFusion)
oFGM440	ATGCGGCCGCAAGCTTTAGTTAGGCAAGTTATCAACTCTC	*hpt*(1-89)::N-I insert, REV (InFusion)
oFGM441	AAGGAGATATACATATGATTAAAATTGCTACAAGAAAG	I-C::*hpt*(90-341) insert, FW (InFusion)
oFGM442	CAGCAGCCTAGGTTATCATTCCTTAGCTCTTGGTC	I-C::*hpt*(90-341) insert (InFusion) and genotyping of Hyg_C, REV
oFGM574	AGGAGATATACCATGGTCACCGTGTTTGGGATTTTG	*sul*(1-125)::N-I insert, FW (InFusion)
oFGM575	ATGCGGCCGCAAGCTTCAATTTGGCAAGTTGTCCACCC	*sul*(1-125)::N-I insert, REV (InFusion)
oFGM576	AAGGAGATATACATATGATCAAAATTGCAACACGGAAGTA	I-C::*sul*(126-278) insert, FW (InFusion)
oFGM577	CAGCAGCCTAGGTTATCAGGCATGGTCTAACCCCCTGTCCCT	I-C::*sul*(126-278) insert, REV (InFusion)
oFGM583	AGGAGATATACCATGGTAACGGTGTTTGGAATACTC	full-length *sul* insert, FW (InFusion)
oFGM584	ATGCGGCCGCAAGCTTCATGCATGATCGAGGCCACG	full-length *sul* insert, REV (InFusion)
oFGM585	AGGAGATATACCATGATTGAACAAGATGGATTGCAC	full-length *nptII* insert, FW (InFusion)

oFGM586	ATGCGGCCGCAAGCTTCAGAAGAACTCGTCAAGAAG	full-length *nptII* insert, REV (InFusion)
oFGM587	CAGCAGCCTAGGTTATCAGAAGAACTCGTCAAGAAG	I-C::*nptII*(192-265) insert, REV (InFusion)

Supplementary Table 2. List of PCR primers used for genotyping.

**Name**	**Sequence (5´ → 3´)**	**Use**
oFGM520	TTGCCAATAGGCAGTAGTCC	Sul_N genotyping, REV
oFGM521	ATCAATCTGTCAGCAGCAAC	Sul_C genotyping, REV
oFGM523	TAGTTTCTAGTTTGTGCGATCG	Genotyping of all constructs containing pUBQ10, FW
oFGM533	TCGATGCGATGTTTCGCTTG	Kan_N and Kan_full genotyping, REV
oFGM442	CAGCAGCCTAGGTTATCATTCCTTAGCTCTTGGTC	I-C::*hpt*(90-341) insert (InFusion) and genotyping of Hyg_C, REV
oFGM444	AGAGACTCAGAGAATTCACC	Hyg_N genotyping, REV
oFGM552	GAGGTAAACCTAGTGCACTC	Sul_full genotyping, REV
oFGM578	AATTATGATCTCTTTCAACTCCG	Kan_C genotyping, REV

Supplementary Table 3. Primer combinations used for genotyping by PCR.

Amplicon	PCR primer combination
Hyg_N	oFGM523 + oFGM444
Hyg_C	oFGM523 + oFGM442
Hyg_full	oFGM523 + oFGM444
Kan_N	oFGM523 + oFGM533
Kan_C	oFGM523 + oFGM578
Kan_full	oFGM523 + oFGM533
Sul_N	oFGM523 + oFGM520
Sul_C	oFGM523 + oFGM521
Sul_full	oFGM523 + oFGM552

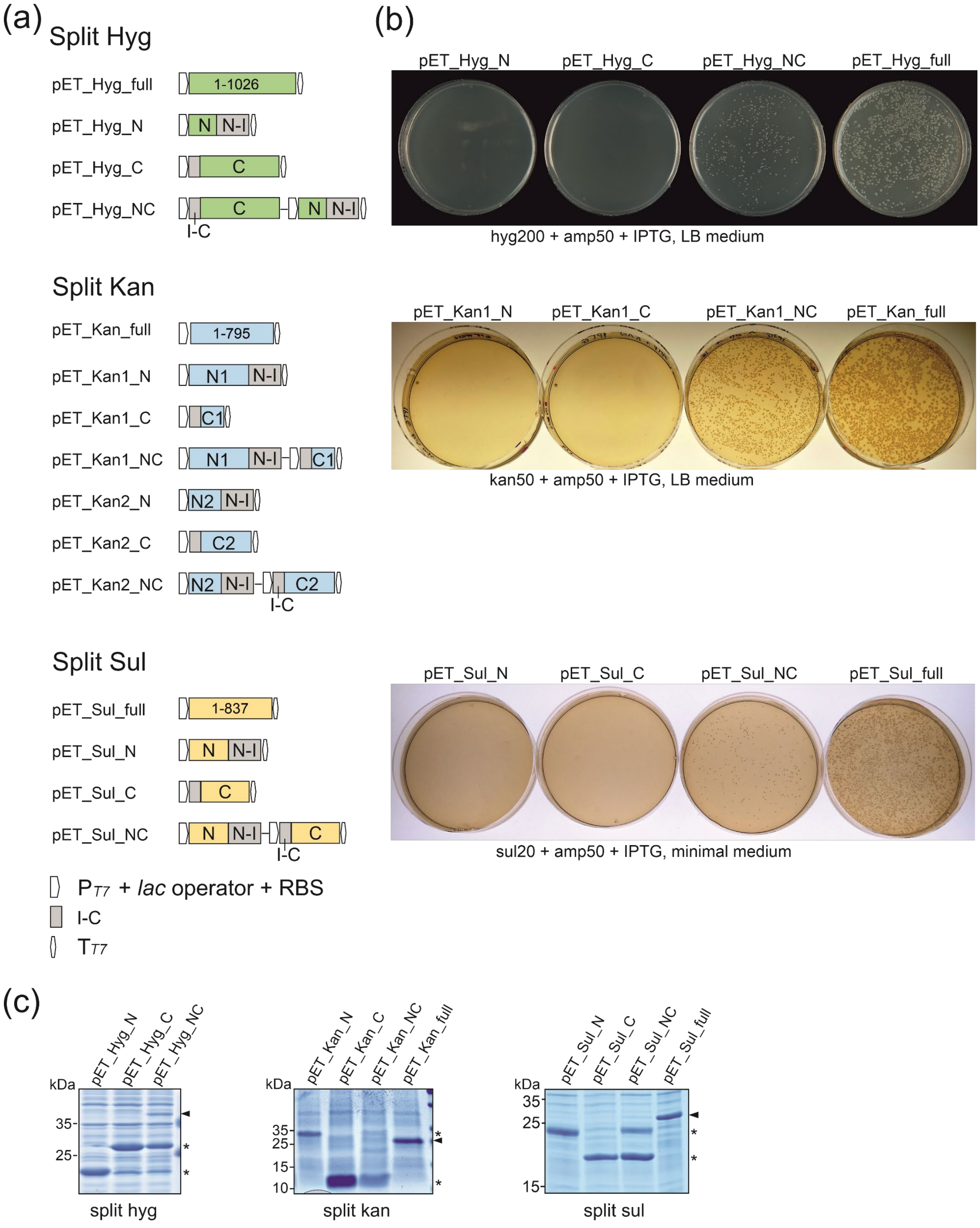

Supplementary Figure 1. Analysis of the three split marker genes in *Escherichia coli*. (**a**) Constructs used for bacterial transformation (cf. Figure 1a and text for details). P_{T7}, phage T7 RNA polymerase gene promoter; T_{T7}, phage T7 RNA polymerase gene terminator; RBS, ribosome-binding site. (**b**) Antibiotic resistance assays of bacterial strains transformed with the various constructs (see text and Supplementary Methods for details). (**c**) Analysis of protein splicing in *E. coli*. A comparison of strains

expressing only the N-terminal or the C-terminal marker protein fragment with a strain co-expressing the two fragments is shown. Total bacterial protein was separated by SDS-PAGE and the gels were stained with Coomassie Brilliant Blue. The expressed marker gene fragments are indicated by asterisks, the full-length proteins, either translated as such or reconstituted by trans-splicing, are marked by arrowheads. See text and Supplementary Methods for details.

(a)

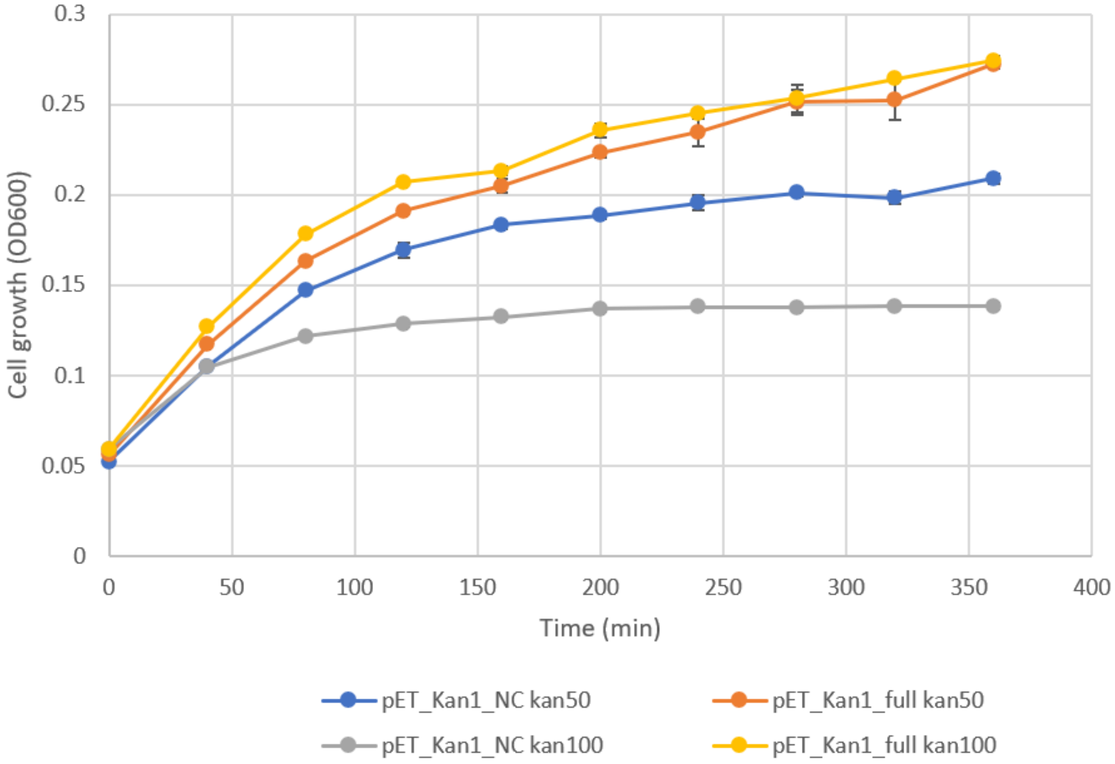

(b)

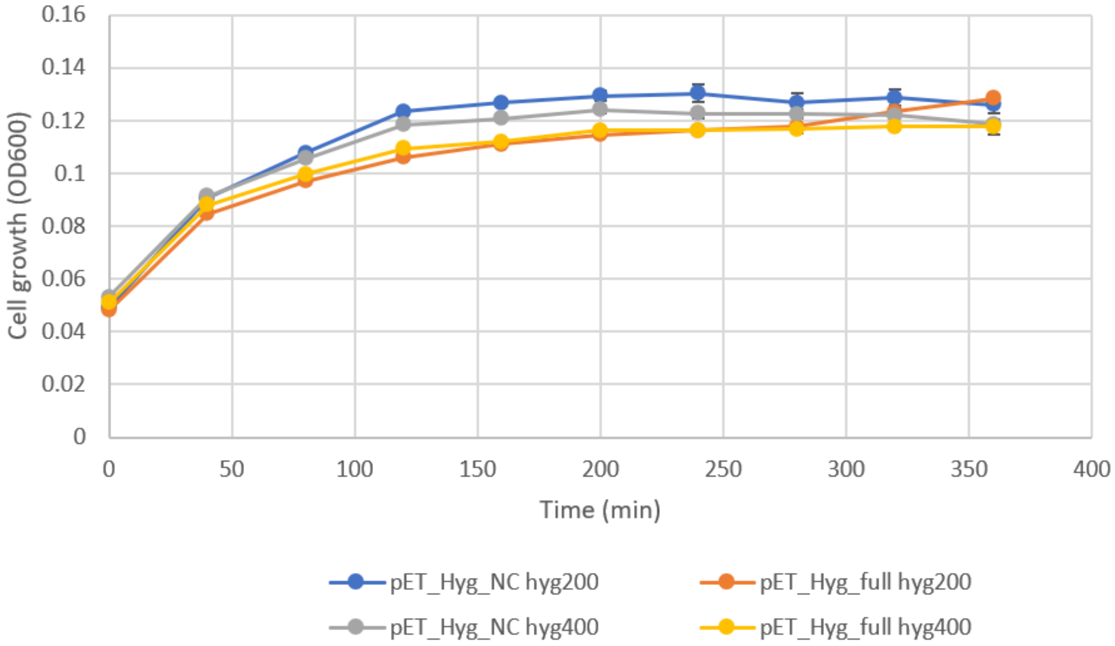

Supplementary Figure 2. Comparative growth assays of *Escherichia coli* strains expressing the split kanamycin and hygromycin markers. Bacterial cultures were

inoculated in LB medium supplemented with ampicillin (50 mg/L) and the indicated concentrations of kanamycin or hygromycin. Following induction of marker expression with IPTG, bacterial growth was monitored for 6 hours. (**a**) Comparison of the split kanamycin resistance gene *nptII* with the full-length *nptII* gene. Note the delayed growth of the cultures expressing the split gene, consistent with the poor performance of the split *nptII* marker in both plants and bacteria (cf. Figure 1 and Supplementary Figure 1). (**b**) Comparison of the split hygromycin resistance gene *hpt* with the full-length *hpt* gene. Similar growth of all cultures confirms the high efficiency of the split *hpt* marker in both plants and bacteria.
